# Outcomes of Noninvasive Positive Pressure Ventilation via RAM Cannula in Pediatric Intensive Care: A Preliminary Report

**DOI:** 10.1155/ijpe/8130440

**Published:** 2026-06-23

**Authors:** Humaira Mustafa, Muhammad Uzair, Sidra Khan, Qalab Abbas

**Affiliations:** ^1^ Pediatric Critical Care Medicine, Department of Pediatrics & Child Health, Aga Khan University Hospital, Karachi, Pakistan, aku.edu; ^2^ Department of Pediatrics & Child Health, Aga Khan University Hospital, Karachi, Pakistan, aku.edu

**Keywords:** NIPPV outcomes, noninvasive positive pressure ventilation, RAM cannula, respiratory failure

## Abstract

**Background:**

Experience in the use of noninvasive positive pressure ventilation (NIPPV) via RAM cannula in the Pediatric Intensive Care Unit (PICU) is limited.

**Objective:**

The objective of this study is to describe the characteristics, outcomes, and complications of patients receiving NIPPV via RAM cannula in our PICU.

**Methods:**

A 12‐month (Jan–Dec 2024) retrospective cohort study included 60 children (1 month–18 years) who received NIPPV via RAM cannula. Patients undergoing elective intubation, with do‐not‐resuscitate orders or with incomplete records, were excluded. Initial NIPPV settings were PEEP 5–12 cm H_2_O, inspiratory pressure 15–28 cm H_2_O, and FiO_2_ titrated to maintain SpO_2_ ≥ 92%. NIPPV failure was defined as escalation to invasive ventilation. Data were analyzed using STATA.

**Result:**

A total of 60 were included with a median age of 15.6 months (IQR 7–41.5). Admitting diagnosis were respiratory illnesses (36; 60%), cardiac disease (9; 15%), and sepsis with MODS (6; 10%). NIPPV indications include respiratory distress in 42 (70%) patients and postextubation support in 18 (30%) patients. Success was achieved in 44 patients (73.3%). Failure was associated with comorbidities (adjusted OR 0.024, *p* = 0.010), higher PRISM III scores (adjusted OR 0.745, *p* = 0.008), higher PEEP (9.8 vs. 8.4 cm H_2_O, *p* = 0.018), and FiO_2_ (90.6% vs. 60.9%, *p* < 0.001). A higher SF ratio was associated with success (189 vs. 136, *p* < 0.001). Failures had more pneumothorax (31% vs. 2.3%, *p* = 0.001), longer PICU stay. (18.3 vs. 8.9 days, *p* < 0.001), and 50% mortality rate, with no deaths among successful cases.

**Conclusion:**

NIPPV is an effective respiratory support modality in the PICU. Predictors of success are lower illness severity, no comorbidities, and better oxygenation.

## 1. Introduction

Respiratory support is one of the leading reasons for Pediatric Intensive Care Unit (PICU) admissions, particularly in low‐ and middle‐income countries (LMICs), where respiratory infections and other conditions are highly prevalent [1]. Lower respiratory infections (LRIs) remain a significant global health burden for children under 5 years. In 2021 alone, LRIs accounted for over 500,000 deaths in this age group, with children under the age of five contributing approximately 13.3% of global LRI mortality [2]. The predominant causes of severe respiratory illness in the pediatric population include asthma, bronchiolitis, pneumonia, and sepsis [3, 4]. A multicenter prospective cohort study reported an incidence density of acute respiratory failure (ARF) of 41.7 cases per 100 person‐years (95% CI: 37.3–47.7), with 75.6% of patients requiring oxygen therapy at admission and a significant proportion requiring high‐flow (36.8%) or low‐flow (29.5%) oxygen systems, and 9.28% needing invasive mechanical ventilation [4]. A local study also found a high need for mechanical ventilation (50.7%) in PICU settings [5], highlighting the substantial burden of respiratory support in this population.

In response to this burden, noninvasive ventilation (NIV) has increasingly become a valuable strategy to reduce intubation rates. It also improves patient comfort and reduces complications, such as ventilator‐associated pneumonia and airway injury [6]. The main modes of noninvasive‐assisted ventilation include noninvasive positive pressure ventilation (NIPPV) and high‐flow nasal cannula (HFNC) [7]. NIPPV delivers positive pressure via nasal or oronasal interface with adjustable positive end‐expiratory pressure (PEEP), inspiratory airway pressure (Pinsp), and fractional inspired oxygen (FiO_2_), thereby improving functional residual capacity (FRC), oxygenation, and CO_2_ elimination [8–11]. Prophylactic NIPPV has been shown to reduce reintubation and pulmonary complications, particularly in high‐risk children [12]. The combination of NIPPV and RAM cannula has shown success in treating conditions such as bronchiolitis, respiratory distress syndrome, and postextubation support in infants and children [8]. This modality is especially valuable in neonatal and PICU settings, where the goal is to optimize respiratory support while minimizing complications. Compared with conventional nasal prongs, the RAM cannula provides more effective pressure transmission (60%–70% with proper fit) and is associated with reduced nasal trauma and shorter support duration in neonates [13, 14].

In our PICU, NIPPV via RAM cannula is utilized as an escalation from HFNC or CPAP and for postextubation support in high‐risk patients. Despite its increasing use, literature describing its outcomes in pediatric intensive care settings remains limited. We aim to describe the outcome of NIPPV via RAM cannula use in children admitted in PICU, and to identify demographic data, including age, diagnoses, and indications for NIPPV, its duration of use, and complications.

## 2. Method

This retrospective cohort study was conducted in our 12‐bed multidisciplinary PICU from January 2024 to December 2024, after approval from the institutional ethical review committee (ERC# 2025‐11160‐33554). The requirement for informed consent was waived due to the retrospective nature of the study. All children aged 1 month to ≤ 18 years, admitted to the PICU, and received NIPPV via RAM cannula during their PICU stay, were included. Patients were excluded if they underwent elective intubation for procedures, had incomplete records, were designated with do‐not‐resuscitate (DNR) status, or were undergoing withdrawal of life support.

In this study, the term NIPPV refers to ventilator‐delivered noninvasive respiratory support provided via RAM cannula using a pressure‐controlled mode with a set inspiratory pressure above PEEP and a backup respiratory rate. Ventilation was delivered using PICU mechanical ventilators capable of noninvasive modes without leak compensation. Inspiratory pressure values represent total peak inspiratory pressure (PIP) settings on the ventilator and not pressure support above PEEP. Delivered tidal volumes were not directly measured due to known interface leak and internal resistance characteristics of the RAM cannula.

Initial ventilator settings typically included a PIP ranging from 15 to 28 cm H_2_O and PEEP between 5 and 12 cm H_2_O, with the backup respiratory rate adjusted according to age and clinical condition. Fraction of inspired oxygen (FiO_2_) was titrated to maintain SpO_2_ ≥ 92%. RAM cannula size selection followed manufacturer weight‐based and nares‐occlusion recommendations to optimize pressure transmission while minimizing nasal trauma.

NIPPV was initiated at the discretion of the attending PICU physician, generally for patients presenting with respiratory distress, defined by tachypnea, increased work of breathing, or hypoxemia and SpO_2_/FiO_2_ (S/F) ratio below 250, persisting despite low flow oxygen therapy, Bubble CPAP, or HFNC, consistent with pediatric NIV literature and pediatric acute respiratory distress syndrome (PARDS) consensus guidance [15–17]. The diagnosis of PARDS was based on the Pediatric Acute Lung Injury Consensus Conference (PALICC‐2) criteria [15]. In our unit, HFNC was preferred for mild to moderate distress, whereas NIPPV was selected for more significant respiratory distress, not requiring immediate intubation.

Effectiveness of NIPPV was defined as improvement in clinical parameters, including normalization or reduction in respiratory rate, reduction in work of breathing (retractions, nasal flaring, grunting), improvement in oxygenation (SpO_2_ ≥ 92% on FiO_2_ < 40%), and stabilization of hemodynamics without escalation of care.

NIPPV failure was defined as escalation to invasive mechanical ventilation due to worsening respiratory distress, persistent or worsening acidosis, hemodynamic instability, or neurological deterioration. Objective physiologic markers including S/F ratio, PRISM III score, ventilatory requirements, and presence of acidosis were analyzed as predictors of failure. Adverse events monitored included pneumothorax confirmed radiologically, abdominal distension associated with feeding intolerance, and nasal injury defined as skin breakdown or pressure‐related trauma at the cannula interface site.

Demographic, clinical, and outcome data were extracted from patient charts and electronic health records. Variables included patient demographics (age, sex, and underlying diagnoses), indications and duration of NIPPV use, vital signs, and laboratory parameters at initiation and during therapy. Outcomes assessed included success or failure of NIPPV, length of PICU stay, survival to discharge, and adverse events. Statistical analysis was performed using STATA. Continuous variables were expressed as means with standard deviations or medians with interquartile ranges, as appropriate, whereas categorical variables were expressed as frequencies and percentages. Comparisons between successful and failed NIPPV groups were performed using *t*‐tests for continuous variables and chi‐square for categorical variables. Given the limited number of failure events (*n* = 16), multivariable logistic regression was performed with cautious variable selection to reduce the risk of overfitting, and findings were interpreted conservatively. A *p* value of < 0.05 was considered statistically significant.

## 3. Results

**Figure 1:**
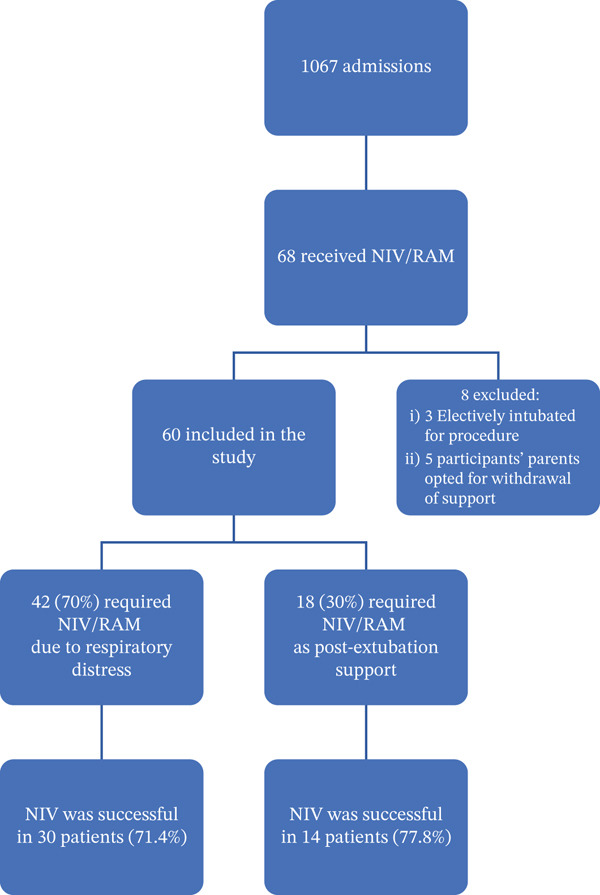
Flow diagram of study population.

During the study period, a total of 1067 patients were admitted to the PICU. Of these, 68 (6.37%) received NIPPV via RAM cannula. Eight patients were excluded, three underwent elective intubation for procedures, and five were withdrawn from ventilatory support based on parental decision, disease severity, and guarded prognosis. Consequently, 60 patients were included in the final analysis (Figure 1).

The median age of the study population was 15.5 months (IQR: 7–41.5) and 41 (68.3%) were male. The primary admission diagnoses were respiratory conditions in 36 (60%), cardiac disease in 9 (15%), sepsis with multiorgan dysfunction syndrome (MODS) in 6 (10%), postoperative care in 6 (10%), and central nervous system (CNS) disease in 2 (3.3%) (Table 1). The median PRISM III score was 4 (IQR: 2–13.5). Comorbidities were present in 28 (46.7%) patients, most commonly neurological (7; 18%), respiratory (6; 21%), and hematological/oncological disorders (6; 21%), followed by genetic/metabolic (5; 25%), cardiovascular (3; 11%), and postliver transplant status (1; 4%).

**Table 1:** Basic demographic and clinical characteristics of study population.

Basic demographic and clinical characteristics	Total (*N* = 60)/%
**Age in months (median with IQR)**	15.5 (7–41.5)
**Gender** (male)	41 (68.33%)
**Admitting diagnosis**	
Respiratory	36 (60%)
Cardiac	9 (15%)
Sepsis with MODS	6 (10%)
Postoperative	6 (10%)
Hemorrhagic shock	1 (1.67%)
Central nervous system	2 (3.33%)
**Comorbidity**	**28 (46.7%)**
**PRISM III score (median with IQR)**	**4 (2–13.5)**
**Indication for NIPPV**	
Respiratory distress	42 (70%)
Postextubation	18 (30%)
**PICU support and therapies used**	
Bronchodilator	55 (91.67%)
Steroids	44 (73.33%)
Diuretics	59 (98.33%)
Sedation	60 (100%)
**NIPPV parameters details**	
Duration of NIPPV use (hours) (median with IQR)	48 (27.5–87.5)
Peak PEEP (mean ± SD)	8.76 ± 2.10
Peak pressure control (mean ± SD)	20.73 ± 3.74
Peak FiO_2_ (%) (mean ± SD)	68.83 ± 20.57
S/F ratio (mean ± SD)	174.93 ± 45.51
**NIPPV success**	**44 (73.3%)**
**Reintubation in postextubation (** **n** = 18 **)**	**4 (22.2%)**
**Time to failure (in hours) median with IQR**	**29.5 (4–65)**
**Survival**	**52 (86.6%)**
**Duration of hospital stay (days) (median with IQR)**	**12 (8.5–20.5)**
**Duration of PICU stay (days) (median with IQR)**	**7.5 (6–12.5)**
**Adverse events**	
Nasal injury	0 (0%)
Abdominal distension	1 (1.67%)
Pneumothorax	6 (10%)

*Note:* Bold values represent different demographic variables.

Abbreviations: FiO_2_, fraction of inspired oxygen; MODS, multiple organ dysfunction syndrome; NIPPV, noninvasive positive pressure ventilation; PEEP, positive end‐expiratory pressure; PRISM, Pediatric Risk of Mortality III.

NIPPV was initiated for respiratory distress in 42 (70%) patients and for postextubation support in 18 (30%) patients. Among those with respiratory distress, 35 (58.3%) required escalation from HFNC due to persistent hypoxemia or increased work of breathing. Common PICU therapies included bronchodilators (55; 91.7%), steroids (44; 73.3%), diuretics (59; 98.3%), and sedation (60; 100%). The mean highest PEEP was 8.76 ± 2.10 cm H_2_O, peak pressure control was 20.73 ± 3.74 cm H_2_O, and mean peak FiO_2_ was 68.83*%* ± 20.57*%*. The mean S/F ratio was 174.93 ± 45.51, which is below 250, threshold defined by PALICC‐2 (2023) for children on nasal NIPPV or HFNC. Based on these criteria, the majority of our patients fell within the range of at‐risk or possible PARDS.

NIPPV was successful in 44 patients (73.3%). Among the 18 patients (30%) who received NIPPV/RAM as postextubation support, 14 (77.8%) were successfully managed without the need for reintubation, whereas 4 (22.2%) required reintubation. The remaining 42 patients (70%) were initiated on NIPPV/RAM for respiratory distress, of whom 30 (71.4%) achieved successful outcomes. The median time to NIPPV failure was 29.5 h (IQR: 4–65). Overall, 52 patients (86.6%) were discharged from hospital. The median duration of NIPPV use was 48 h (IQR: 27.5–87.5). The median duration of hospital stay was 12 days (IQR: 8.5–20.5), and the median PICU stay was 7.5 days (IQR: 6–12.5). Adverse events were pneumothorax in six patients (10%), and abdominal distension in one (1.67%).

**Figure 2:**
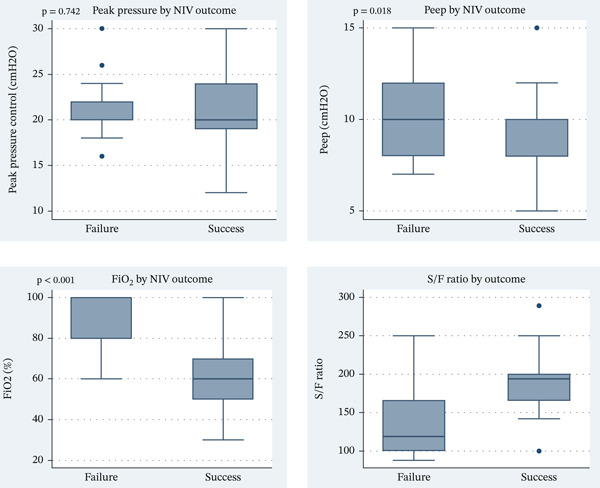
Peak pressure, PEEP, FiO_2_, and SF ratio comparison in NIPPV success and failure.

Comorbidities were observed in 68.75% of patients in the failure group compared with 38.6% in the success group (*p* = 0.039). The peak PEEP was 9.81 cm H_2_O in the failure group and 8.38 cm H_2_O in the success group (*p* = 0.018), and the FiO_2_ requirement was 90.63% in the failure group and 60.90% in the success group (*p* < 0.001). The SF ratio was 135.62 in the failure group and 189.22 in the success group (*p* < 0.001) (Figure 2). Pneumothorax occurred in 31.3% of patients in the failure group compared with 2.3% in the success group (*p* = 0.001). The mean PICU length of stay was 8.9 days (95% CI: 6.92–10.90) in the success group and 18.31 days (95% CI: 11.36–25.26) in the failure group (*p* < 0.001). The mean hospital length of stay was 14.59 days (95% CI: 11.02–18.16) in the success group and 21.87 days (95% CI: 14.76–28.98) in the failure group (*p* = 0.045). Mortality was recorded in 8 of 16 patients (50%) in the failure group, whereas no deaths occurred in the success group (*p* < 0.001) (Table 2).

**Table 2:** Comparison of clinical characteristics and outcomes between NIPPV success and failure groups.

Variable	NIV success (*n* = 44)	NIV failure (*n* = 16)	*p*value
Age (mean ± 95% CI)	28.94 (16.10–41.78)	37 (21.14–52.86)	0.483
PRISM III (mean ± 95% CI)	6.09 (4.16–8.024)	9.25 (5.26–13.24)	0.110
Comorbidity	17 (38.63%)	11 (68.75%)	0.039
Length of stay in PICU (days) (mean ± 95% CI)	8.9 (6.92–10.90)	18.31 (11.36–25.26)	< 0.001
Length of stay in hospital (days) (mean ± 95% CI)	14.59 (11.02–18.16)	21.87 (14.76–28.98)	0.045
Diagnosis			
CVS	7 (15.9%)	2 (%)	0.744
CNS	2 (4.54%)	0 (%)	0.386
Respiratory	28 (63.63%)	8 (%)	0.340
Sepsis with MODS	4 (9.08%)	2 (%)	0.697
Hemorrhagic shock	0 (0%)	1 (%)	0.094
Post‐op	3 (6.81%)	3 (%)	0.173
Adverse effects			
Nasal injury	0 (0%)	0 (0%)	—
Abdominal distension	0 (0%)	1 (6.25%)	0.094
Pneumothorax	1 (2.27%)	5 (31.25%)	0.001
NIV duration (hours) (mean ± 95% CI)	62.405 (51.34–73.56)	48.37 (18.05–78.70)	0.264
Indication of NIPPV			
Postextubation	14 (31.38%)	4 (25%)	0.610
Respiratory distress	30 (68.18%)	12 (75%)	0.610
NIPPV Parameters			
Peak PEEP (mean ± SD)	8.38 (7.80–8.96)	9.81 (8.60–11.03)	0.018
Peak pressure control (mean ± SD)	20.63 (19.45–21.82)	21.00 (19.17–22.82)	0.742
Peak FiO_2_ (%) (mean ± SD)	60.90 (56.02–74.14)	90.63 (82.49–98.75)	< 0.001
S/F ratio (mean ± SD)	189.22 (178.15–200.30)	135.62 (111.22–160.02)	< 0.001
Mortality (*n* = 8)	0 (0%)	8 (50%)	< 0.001

*Note:* Continuous variables were compared using the independent samples *t*‐test (presented as mean ± SD or mean with 95% CI), whereas categorical variables were analyzed using the chi‐squared test. A *p* value < 0.05 was considered statistically significant.

Abbreviations: FiO_2_, fraction of inspired oxygen; MODS, multiple organ dysfunction syndrome; NIPPV, noninvasive positive pressure ventilation; PEEP, positive end‐expiratory pressure; PRISM III, Pediatric Risk of Mortality III; S/F ratio, SpO_2_/FiO_2_ ratio.

The presence of comorbidity reduced the likelihood of success (adjusted OR: 0.024; 95% CI: 0.001–0.41; *p* = 0.010). Higher PRISM III scores were also negatively associated (adjusted OR: 0.745; 95% CI: 0.60–0.93; *p* = 0.008). Lower peak FiO_2_ was a strong predictor of success (adjusted OR: 0.87; 95% CI: 0.81–0.94; *p* = 0.001). Shorter PICU stay (OR: 0.905; *p* = 0.004), higher SF ratio (OR: 1.04; *p* < 0.001), and lower peak PEEP (OR: 0.715; *p* = 0.029) were also associated with successful NIPPV outcomes. The age of 5–18 years was associated with lower odds of success in unadjusted analysis (OR: 0.16; *p* = 0.034) but did not remain significant in the adjusted model (Table 3).

**Table 3:** Logistic regression table adjusted for success of NIPPV (*n* = 60).

	Unadjusted		Adjusted	
	OR (95% CI)	*p*value	OR	*p*value
Age in years				
< 1 year	1			
1–5 years	0.48 (0.12–1.94)	0.308		
5–18 years	0.16 (0.03–0.87)	0.034		
Gender				
Male	1			
Female	1.03 (0.30–3.52)	0.967		
Length of stay in PICU (days)	0.905 (0.85–0.97)	0.004		
Hospital stay (days)	0.956 (0.91–1.00)	0.070		
SF ratio	1.04 (1.02–1.06)	< 0.001		
Comorbidity	0.286 (0.08–0.97)	0.044	0.024 (0.001–0.41)	0.010
PRISM score	0.936 (0.862–1.02)	0.115	0.745 (0.60–0.93)	0.008
Duration of NIPPV ventilation	1.01 (0.99–1.02)	0.263		
Indication of NIPPV				
Postextubation	1.4 (0.38–5.12)	0.611		
Escalation of support	1.58 (0.501–5.03)	0.431		
Respiratory distress	0.71 (0.19–2.61)	0.611		
Parameters				
Peak PEEP	0.715 (0.53–0.96)	0.029		
Peak pressure control	0.97 (0.84–1.13)	0.738		
Peak FiO_2_ (%)	0.91 (0.87–0.95)	< 0.001	0.87 (0.81–0.94)	0.001
Diagnosis				
CVS	1.32 (0.24–7.16)	0.744		
Respiratory	1.75 (0.55–5.56)	0.343		
Sepsis with MODS	0.7 (0.12–4.25)	0.698		
Post‐op	0.32 (0.06–1.77)	0.190		
Therapies				
Steroids	0.89 (0.24–3.30)	0.860		
Bronchodilator	1.95 (0.29–12.91)	0.488		

Table 4 summarizes the causes of NIPPV failure. Multiple factors were present in several patients.

**Table 4:** Causes of NIPPV failure (*n* = 16).

Major cause	Number of patients (*n*)	Percentages (%)
Respiratory/metabolic acidosis	6	37.5%
Severe ARDS	5	31.3%
Low GCS/neurological causes	3	18.8%
Infections (HAI)	3	18.8%
Hemodynamic instability/MODS	3	18.8%
Secretion retention/lung collapse	2	12.5%

*Note:* Some patients had more than one contributing factor.

## 4. Discussion

In this pilot study, we observed an overall success rate of NIPPV of 73.3% and survival to discharge of 86.6%. However, this success was strongly influenced by baseline illness severity and physiologic status, suggesting that RAM‐based NIPPV should not be interpreted as universally effective across all PICU admissions. Rather than broadly endorsing this modality for all PICU patients, our findings help delineate the clinical context in which RAM‐based NIPPV appears most beneficial.

The success rate observed in our cohort aligns with previously reported outcomes of NIPPV in PICU settings, where success generally ranges from 66% to 75% depending on the interface, mode, and underlying disease of study population [6, 18–20]. However, most prior data are derived from studies using masks, nasal prongs, or other interfaces with different modes of NIV, and are therefore not directly comparable. Evidence specifically addressing NIPPV via RAM cannula in the PICU remains limited [6, 18, 20]. Outpatient series in children with chronic disease have demonstrated feasibility and higher success rates (85%–94%) [8, 9], but these represent highly selected populations rather than critically ill PICU patients. Thus, our study contributes novel evidence by evaluating RAM cannula NIPPV in a broader, mixed‐acuity pediatric cohort, bridging the gap between outpatient experiences and disease‐specific studies.

The median age in our population was 15.6 months (IQR 7–41.5 months), representing infants and young children. This age group is particularly vulnerable to respiratory failure, making noninvasive strategies especially relevant. Similar age distributions have been described in previous pediatric cohorts [6, 18, 19], whereas neonatal‐focused trials [21, 22] limit comparability with our older population. Thus, our study provides important insights in a postneonatal pediatric population. Underlying diseases were predominantly respiratory, but there were patients with other diseases provided NIPPV, which may reflect lack of standard guidelines and learning curve on this disease‐specific respiratory support. Our results suggest that NIPPV is less suitable in children with shock, hemodynamic instability, or low GCS. The main causes of failure were acidosis (37.5%), severe ARDS (31.3%), neurological compromise (18.8%), infections (18.8%), MODS/hemodynamic instability (18.8%), and secretion retention or lung collapse (12.5%), with overlap in many cases. This pattern indicates that NIPPV via RAM cannula may be most appropriate in moderate‐severity respiratory failure, whereas children with advanced organ dysfunction or severe lung injury may warrant earlier consideration of invasive ventilation [15, 23, 24]. Overall, these findings suggest that patient selection is critical.

Predictors of failure in our cohort included higher ventilator pressures and worse oxygenation indices, consistent with previous reports demonstrating that lower S/F ratio, higher PEEP and FiO_2_ requirements, and comorbidities increase the likelihood of intubation [12, 24]. Similar findings have been described in postcardiac surgery populations, where metabolic acidosis and higher severity indices predict poorer outcomes [11, 20, 25].

Importantly, our cohort included both primary respiratory distress and postextubation support populations. Although success rates were comparable between subgroups, the physiologic mechanisms underlying failure differ. In primary respiratory failure, acidosis and severe PARDS were dominant contributors [23, 24], whereas postextubation failure was often related to underlying disease severity and secretion burden [18, 26]. Because these groups represent distinct physiologic entities, pooling them introduces clinical heterogeneity and reinforces the need for careful patient selection rather than uniform application of RAM‐based NIPPV. Future studies should evaluate these groups separately.

In the postextubation subgroup, reintubation occurred in approximately 22%, which is similar to previous pediatric studies using NIPPV or HFNC after extubation [10, 18]. These results support the role of NIPPV via RAM as an effective bridge for high‐risk extubation [26] while emphasizing the need for vigilant monitoring in patients at risk of failure. Mortality was significantly higher among NIPPV failures (50%), with no deaths in the success group, underscoring the importance of early risk stratification and timely escalation to invasive ventilation when necessary.

The use of higher PEEP and FiO_2_ in the failure group likely reflects underlying disease severity rather than causation. Given the partial pressure transmission and intrinsic resistance characteristics of the RAM cannula interface [13, 14], set inspiratory pressures may not directly correspond to delivered alveolar pressures, and therefore, escalation of ventilator settings should be interpreted cautiously. However, the higher pneumothorax observed rate in our study (10%) among failures suggests that escalation of pressures in poorly compliant lungs may increase barotrauma risk [15, 24]. Clinicians should therefore exercise caution when escalating inspiratory pressures, particularly in severe PARDS [15, 24], where patient self‐inflicted lung injury may also contribute [27].

The absence of nasal trauma in our cohort supports prior evidence that RAM cannula may reduce interface‐related complications compared with traditional masks or prongs [16, 20]. This feature may be particularly advantageous in resource‐limited settings where patient comfort, tolerance, and nursing workload are critical considerations.

This study evaluates RAM cannula–based NIPPV in a real‐world multidisciplinary PICU, reflecting routine clinical practice across a heterogeneous patient population. The inclusion of both children with primary respiratory failure and those receiving postextubation support enhances the clinical applicability of the findings. In addition, the systematic analysis of objective physiologic markers, including the S/F ratio and PRISM III score, allowed identification of meaningful predictors of NIPPV failure and provides practical insight into patient selection for noninvasive respiratory support.

This study has several limitations. First, its retrospective single‐center design may limit generalizability. Second, ventilator settings were not standardized and were adjusted according to physician discretion, which may introduce variability in treatment delivery. Third, the relatively small sample size limited the ability to perform detailed age‐stratified or disease‐specific subgroup analyses. Additionally, escalation to invasive ventilation was used as the primary definition of failure, which may not fully capture the dynamic physiologic trajectory preceding clinical deterioration.

Future multicenter prospective studies are needed to compare RAM cannula with other noninvasive interfaces, define safe and effective pressure thresholds, and refine predictors of NIPPV failure. Incorporating serial physiologic monitoring, including oxygenation indices such as the S/F ratio and objective assessments of work of breathing, may provide more granular insight into early predictors of deterioration and help guide timely escalation of respiratory support.

Overall, our findings suggest that the optimal role of RAM cannula–based NIPPV may be in hemodynamically stable children with moderate respiratory distress, preserved neurological status, and in selected high‐risk patients requiring postextubation respiratory support. Conversely, caution is warranted in children with severe PARDS, significant metabolic acidosis, shock states, or depressed consciousness. These findings highlight the importance of strategic, physiology‐guided patient selection rather than broad endorsement of RAM‐based NIPPV for all critically ill children.

## 5. Conclusion

NIPPV delivered via RAM cannula is increasingly being used in a heterogeneous PICU population, yet clear criteria guiding its application remain limited. Our study adds to the evolving literature by characterizing outcomes and identifying predictors of failure in a mixed‐acuity cohort, highlighting the modality′s strongest performance in younger children and those without significant comorbidities. These findings underscore the need to refine patient selection for RAM‐based NIPPV and to better define thresholds for safe and effective use in ARF and postextubation support. There is an opportunity to develop more unified clinical parameters that incorporate serial physiologic indices and objective markers of disease severity to guide escalation decisions and minimize delays to intubation. Future multicenter prospective studies are warranted to compare RAM cannula with other noninvasive interfaces and to clarify its optimal role within modern pediatric respiratory support strategies.

## Funding

No funding was received for this manuscript.

## Ethics Statement

The study was approved by the Aga Khan University Hospital, Institutional Ethical Review Committee (ERC# 2025‐11160‐33554).

## Conflicts of Interest

The authors declare no conflicts of interest.

## Data Availability

The data that support the findings of this study are available from the corresponding author upon reasonable request.
